# Chemoselective Electrochemical Hydrogenation of Ketones and Aldehydes with a Well‐Defined Base‐Metal Catalyst

**DOI:** 10.1002/chem.202002075

**Published:** 2020-10-04

**Authors:** Igor Fokin, Inke Siewert

**Affiliations:** ^1^ Institut für Anorganische Chemie Universität Göttingen Tammannstr. 4 37077 Göttingen Germany

**Keywords:** catalysis, homogeneous catalysis, molecular electrochemistry, spectroelectrochemistry, reaction mechanisms

## Abstract

Hydrogenation reactions are fundamental functional group transformations in chemical synthesis. Here, we introduce an electrochemical method for the hydrogenation of ketones and aldehydes by in situ formation of a Mn‐H species. We utilise protons and electric current as surrogate for H_2_ and a base‐metal complex to form selectively the alcohols. The method is chemoselective for the hydrogenation of C=O bonds over C=C bonds. Mechanistic studies revealed initial 3 e^−^ reduction of the catalyst forming the steady state species [Mn_2_(H_−1_L)(CO)_6_]^−^. Subsequently, we assume protonation, reduction and internal proton shift forming the hydride species. Finally, the transfer of the hydride and a proton to the ketone yields the alcohol and the steady state species is regenerated via reduction. The interplay of two manganese centres and the internal proton relay represent the key features for ketone and aldehyde reduction as the respective mononuclear complex and the complex without the proton relay are barely active.

## Graphical abstract

**An electrochemical protocol** is herein presented for the hydrogenation of ketones forming selectively the corresponding alcohols using protons and electric current. A dinuclear manganese catalyst is used with an internal proton relay to facilitate the reaction. Investigation by means of electrochemical methods and IR spectroelectrochemistry shed light on the catalytic cycle.

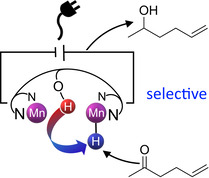

## Introduction

Hydrogenation reactions are of utmost academic and industrial importance as they represent one of the key reactions for the synthesis of bulk chemicals as well of elaborated organic molecules.^[1]^ Initially, research on hydrogenation reactions focused on the use of noble metal complexes based on Ru, Rh, Ir, Pd, Pt and remarkable achievements have been made using such complexes. Recently the focus shifted to the use of non‐precious metal catalysts, as these metals exhibit a higher availability in earth's crust.^[2]^ In the first instance, the direct reduction of C=O bonds with H_2_ yielding the alcohols seems to be a very atom and redox economic approach. However, the reaction protocols for such hydrogenation reactions require often harsh conditions with high H_2_ pressure at elevated temperature. Furthermore, each ton of H_2_ produced via steam reforming produces 11.88 tons of CO_2_ and the process operates with an external energy efficiency of only 60.4 %, and thus, more sustainable protocols are highly desirable.^[3]^

Another approach, yet rarely investigated in molecular catalysis, is the use of protons and electric current as hydride source.^[4]^ Electrochemistry has been used recently more frequently to construct (organic) molecules, as it allows to use energy directly from renewable sources. It represents a versatile tool for more redox, atom and energy economic synthesis.^[5]^ The reactive species can be formed in situ under mild conditions in high local concentration near the electrode surface.^[6]^

**Figure 1:**
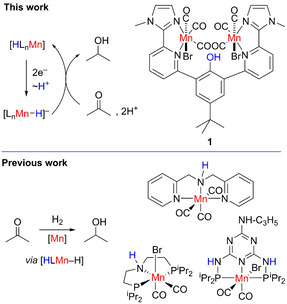
Top: Electrochemical hydrogenation of acetone to isopropanol. Bottom: Selected Mn (pre)catalysts for the hydrogenation of ketones.

In a putative electrochemical hydrogenation protocol using a molecular catalyst, the key M−H species for the catalytic hydrogenation reaction is formed electrochemically upon reduction and protonation of the metal ion (Figure 1, top).

However, such M−H species also represent key intermediates in the electrochemical hydrogen production catalysed by metal complexes, and thus, the in situ formed metal hydride species may react with excess of protons forming H_2_ instead of reacting with the ketone.^[7]^ From a thermodynamic point of view, the hydrogenation, for example, of acetone is thermodynamically favoured over proton reduction due to the hydrogenation free energy (Δ*G* ^0^ ≈−23 kJ mol^−1^, see the Supporting Information for detailed analysis).^[8]^ This is also evident from the solution hydricity of isopropanol, which is larger than the hydricity of H_2_. In other words, hydride transfer from a putative L_*n*_M−H species has a higher driving force for the former reaction than for H^+^ (*cf*. ΔΔ*G* ^0^≈−46 kJ mol^−1^, see the Supporting Information for detailed calculation).^[9]^ However, the barrier is expected to be smaller in the latter case and thus, catalysts are required with large overpotentials for H_2_ evolution or the reaction should be run near the thermodynamic potential. Furthermore, the direct reduction of the ketone forming the ketyl radical must be prevented.[^5b^, ^10^, ^11^]

The electrochemical hydrogenation of ketones and aldehydes with polypyridine rhodium complexes has been investigated previously, though, C=O and C=C bonds were hydrogenated with such systems.[^4a^, ^4b^, ^4c^, ^4d^, ^4e^] Selectivity is also a major issue for protocols using metal or metal modified electrodes, electric current and protons. In such systems, adsorbed H atoms, which are generated electrochemically under acidic conditions, have been proposed as active species.^[12]^ However, chemoselective electrochemical hydrogenation protocols for unsaturated C=O compounds forming the corresponding alcohols are still challenging, as the desired hydrogenation reaction competes with the hydrogenation of the C=C bond, which is thermodynamically favoured (see Supporting Information for calculation), the direct reduction of the ketone forming unselectively the follow up products of the ketyl radical, and the hydrogen evolution reaction (HER).[^12f^, ^12i^, ^13^]

We had reasonable evidence that the binuclear manganese complex **1** may serve as a catalyst in the electrochemically‐driven hydrogenation reaction of C=O bonds (Figure 1). We knew from a previous study on **1** that (i) the proton relay in **1** facilitates the formation of Mn−H species upon electrochemical reduction and protonation, (ii) the Mn−H species reacts with CO_2_ forming formate, and (iii) it shows only very low reaction rate for the H_2_ evolution.^[14]^ Furthermore, **1** exhibits several structural features, which proved to be beneficial in manganese hydrogenation catalysts. The selective hydrogenation of ketones by a manganese complex was reported for the first time in 2016.^[15]^ A few other reports appeared subsequently, and most of the catalysts have in common that they bear a pincer ligand, a basic site in the ligand backbone, and carbonyl ligands at the Mn centre (Figure 1, bottom).[^2g^, ^16^] The bifunctionality of the catalysts is supposed to be critical for catalysis.[^2g^, ^15^, ^16^, ^17^] It promotes the initial, heterolytic cleavage of H_2_ forming the Mn−H species and the proton is accepted by the basic ligand site.

## Results and Discussion

**Figure 2:**
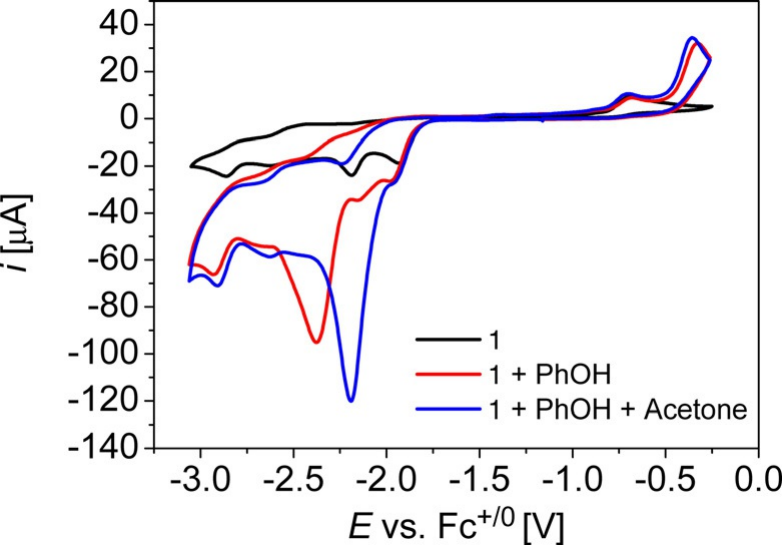
CV data of **1** in THF, *c*_1_=1 mm, *c*_phenol_=10 mm, *c*_acetone_=100 mm, 0.2 m *n*Bu_4_NPF_6_, *ν*=0.1 Vs^−1^.

Complex **1** was synthesised and characterised as described previously.^[14]^ It exhibits several reduction processes, between −1.9 V and −2.9 V, which are coupled by chemical reactions as described previously, and the potentials depend on the solvent (Figure 2, Table 1, Figure S14, Figure S15).^[14]^ All potentials in this study are referenced versus the Fc^+|0^ redox couple and a GC working electrode was used for all CV measurements.

**Table 1:** Peak potentials vs. Fc^+|0^ V^−1^ of the reduction processes of **1** in various solvents, *ν*=0.1 V s^−1^, 0.1 m *n*Bu_4_NPF_6_ (DMF and CH_2_Cl_2_), 0.2 m *n*Bu_4_NPF_6_ (THF), GC electrode.

Solvent	*E*_p,c,1_	*e*_p,c,2_	*E*_p,c,3_	*E*_p,c,4_	*E*_p,c,5_
DMF^[14]^	−2.06	−2.20	−2.40	−2.54	−2.76
THF	−1.94	−2.20	−2.47	−2.63	−2.87
CH_2_Cl_2_	−1.87	−2.14

In order to decrease the driving force for HER as an undesirable side reaction, phenol as weak acid was chosen, since this leads to an estimated standard potential for hydrogen evolution of −2.16 V in THF.^[18]^ Adding 10 equiv of phenol to a solution of **1** (1 mm) led to an increased current at *E*_p,cat_=−2.38 V in THF and at *E*_p,cat_=−2.35 V in CH_2_Cl_2_ (*ν*=0.1 Vs^−1^, Figure 2, Figure S14, Figure S15). Upon adding 100 equiv of acetone to these mixtures, the catalytic waves shift positively, that are to peak potentials *E*_p,cat_=−2.21 V in CH_2_Cl_2_ and *E*_p,cat_=−2.19 V in THF, which is near the peak potential of the second reduction wave in **1**. The peak potential shifts upon adding acetone, indicated different reactivities, and thus, controlled potential electrolysis (CPE) experiments were run to evaluate product formation.

A CPE experiment at −2.2 V in CH_2_Cl_2_ showed indeed the formation of isopropanol in the crude reaction mixture. However, CH_2_Cl_2_ turned out to be not suitable as a solvent, since the current dropped quickly and Faraday efficiency (FE) for isopropanol formation was rather low (≈10 %, Figure S16). The current decreased with the first 20 minutes of the electrolysis and was re‐established after polishing the electrode, which indicates a fouling of the electrode surface under catalytic conditions. A CPE experiment in THF at −2.2 V revealed isopropanol formation with higher FE and the current held more constant (Figure S17). Thus, we focused on THF. We optimised the reaction conditions at first. The reaction must be run in a divided cell and an excess of phenol is necessary to achieve reasonable conversion. Catalyst concentration of 1 mm proved to be ideal in terms of current flow and FE for acetone formation. Reasonable conversion of acetone to isopropanol was accomplished with a carbon foam electrode, 5 mol % catalyst and 7.5 equiv of phenol. In these optimised reaction conditions, H_2_ formation was successfully supressed and counted for only 5 % of the injected charge, whereas 88 % of the charge counted for isopropanol formation. When the ratio of acetone to catalyst was increased, a TON up to 32 was observed, however, the conversion of acetone was lower.

With these optimised conditions in hand, we investigated the substrate scope of the protocol (Figures S17–S32, Table 2). Acetone was hydrogenated with yields of 60 % and FE of 88 %. Next, we explored the influence of the carbon chain lengths. Symmetrical (entry 2) and unsymmetrical ketones (entry 3) with longer chain lengths were successfully hydrogenated, with yields of 65 % and 75 %, respectively. The catalyst and phenol concentration were maintained the same and the currents were similar to the one in experiment in entry 1.

**Table 2:** Overview on the electrosynthesis experiments.

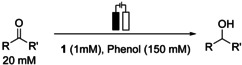				
	Substrate	Product	FE %	Yield^[a]^ %
1	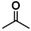	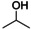	88	60
2	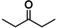	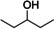	60	65
3	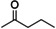	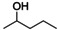	69	75
4	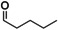	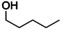	93	99
5	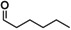	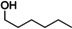	77	90
6	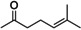	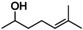	32	35
7	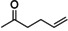	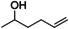	68	65
8	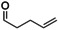	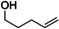	47	40

[a] The yields were determined by ^1^H NMR spectroscopy using the internal standard benzene.

The method is not limited to the hydrogenation of ketones, but also feasible for aldehydes. 1‐Pentanal was hydrogenated with a yield of 99 % under otherwise identical conditions (entry 4). The aldehyde with a longer chain length, that is, 1‐hexanal, was also successfully employed (entry 5).

Lastly, we show that the protocol is selective for C=O bonds over C=C bonds as two unsaturated ketones and one aldehyde were converted chemoselectively. Electrolysis of 6‐methylhept‐5‐en‐2‐one yielded the corresponding alcohol. However, the yield was lower likely due to the steric bulk (entry 6). Terminal double bonds are also tolerated as shown in the hydrogenation reaction of hex‐5‐en‐2‐one forming selectively hex‐5‐en‐2‐ol with yields of 65 % (entry 7). The unsaturated aldehyde pent‐4‐enal, was selectively converted to the alcohol, too (entry 8).

We had no evidence that any of the carbonyl compounds is reduced directly at the electrode surface as none of them is reduced at the potential of the catalysis in the absence of **1** as evident from the CV data (Figure S18, Figure S20, Figure S22, Figure S24, Figure S26, Figure S28, Figure S30, Figure S32).

The selectivity for the hydrogenation of C=O over C=C bonds is similar to what has been displayed for manganese compounds in thermal hydrogenation protocols using H_2_,[^2g^, ^15^, ^16^, ^17^] however, selective electrochemical protocols using base metal complex are unknown to the best of our knowledge. The electrochemical hydrogenation of similar unsaturated and saturated substrates at metal electrodes often suffers from electrochemical efficiency due to simultaneous H_2_ evolution as well as selectivity. Various products are often formed,^[19]^ for example, hydrogenolysis, hydrogenation of the double bond, coupling products of the ketyl radical,^[10]^ cyclisation products for unsaturated carbonyl compounds^[20]^ due to direct reduction of the ketone. However, at Raney‐Nickel electrodes, the selectivity for alcohol formation is reported to be good, though, FE for aliphatic ketones were only moderate.^[21]^ The surface orientation also plays a crucial role for selectivity as recently shown on Pt electrodes. Acetone is reduced nearly selectively to propanol at stepped Pt(111) crystals and to propane at stepped Pt(100) electrodes, which was rationalised by differences in the coordination number of the surface active sites.^[22]^

Next, we investigated the mechanism of the reaction of **1**, acetone and phenol in detail. Acetone is the source for isopropanol, as confirmed by ^13^C isotope labelling studies: when the ^13^C=O‐labelled acetone was used, ^13^C‐OH‐labelled isopropanol was formed. No other ^13^C‐labelled products were observed by ^13^C NMR spectroscopy, which tentatively excludes direct reduction of acetone forming the ketyl‐radical (Figure S3, Figure S4). This was corroborated by CV study of acetone and **1**, in which no catalytic wave was observed (Figure S36) and in a CPE experiment of ^13^C=O‐labelled acetone and phenol without catalyst, in which we do not observe any conversion at all (Figure S3). Thus, we assume that the alcohol is not formed via direct electrochemical reduction of the ketones,[^5b^, ^10^, ^11^] but instead via formation of a transient Mn−H species.

Isotope labelling studies employing [D_6_]‐phenol showed, that the maximum current of the catalytic wave does not change with regard to the same experiment with [H_6_]‐phenol, though, catalysis is not under kinetic control in these experiments (Figure S33, Figure S35).^[23]^ When deuterated phenol was used in a CPE experiment, the current was considerably lower (Figure S34), indicating a normal H/D isotope effect. This tentatively excludes an H‐atom transfer of the putative Mn−H species to the ketone as such reversible HAT to carbon skeletons usually show an inverse isotope effect due to the low initial frequency of the Mn−H/Mn−D bond relative to that of the C−H/C−D bond.^[24]^ CV experiments with varying concentrations of catalyst revealed that the current of the catalytic wave increases linearly with increasing concentrations of **1** (Figure S37). Since the catalytic current is proportional to catalyst's concentration according to Equation 1,^[25]^ this means that the reaction is first order in **1** [Eq. 2]:

(1) $$i_{cat}=n_{cat}\cdot F\cdot A\cdot c_{cat}\cdot \sqrt{D_{cat}\cdot k_{obs}}$$

(2) $$k_{obs}=k_{cat}\cdot c_{PhOH}^{x}\cdot c_{acetone}^{y}$$

*n*_cat_=number of transferred electrons, F=Faraday constant, *k*_obs_=observed or apparent catalytic rate constant, *c*_cat_=catalyst concentration, *c*_PhOH_=phenol concentration, *c*_acetone_=acetone concentration, *x*, *y*=reaction order, *A*=electrode area.

The reaction order in phenol was estimated under pseudo first order conditions with varying amounts of phenol. The current tentatively increases proportional with the square root of the phenol concentration (Figure S38). This means that the catalysis is first order in phenol according to Equations (1) and (2), as the catalytic current *i*_cat_ is proportional to the square root of the apparent rate constants *k*_obs_. This would be in line with one internal proton transfer from the phenol unit to the metal centre forming the Mn−H species, and proton transfer from external phenol. The reaction order in acetone could not be determined. Large excess of PhOH leads to a prominent wave for hydrogen evolution, which decreases with increasing amounts of acetone, indicating that this reaction is supressed successfully with increasing amounts of acetone (Figure S39). However, the redox process assigned to the H_2_ evolution reaction is only ≈180 mV more negative than the catalytic wave for the hydrogenation reaction, and thus reliable determination of the current under large excess of phenol is not possible.

**Figure 3:**
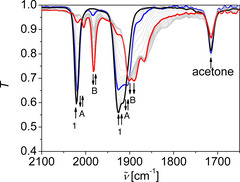
Linear sweep IR‐SEC of **1**, acetone and phenol. The black spectrum is the start spectrum, the blue one that of the species after initial reduction and the red one during catalysis. *c*_1_=3 mm, *c*_phenol_=30 mm, *c*_acetone_=30 mm, 0.2 m *n*Bu_4_NPF_6_, *ν*=0.0025 Vs^−1^, THF.

IR spectroelectrochemical experiments (IR‐SEC) were conducted to get structural information on the in situ formed reduced Mn(CO)_3_ species during catalysis (Figure 3).^[26]^ The redox chemistry of **1** in DMF has been investigated in detail previously by various methods, and the species formed in THF upon reduction are essentially the same (Figure S46).^[14]^ Initial reduction of **1** in the absence of any substrate leads to the formation of **A** (blue trace). The CO stretching frequencies of **A** at 2017, 1923, and 1904 cm^−1^ are characteristic for a dinculear Mn species in which both Mn^I^ ions are coordinated by one X‐type ligand, three facial carbonyl ligands, and the α‐diimine units of L. Thus, **A** was previously assigned to [Mn_2_(H_−1_L)(CO)_3_Br] (H_−1_L denotes the ligand deprotonated at the phenol unit forming a phenolate).^[14]^ That is, initial reduction of **1** induces homolytic O−H bond cleavage, loss of one bromide ligand and coordination of the resulting phenolate to the metal ion. The overall reaction for the formation of **A** from **1** is redox‐neutral for the complex and equal to HBr elimination from **1**, and the proton is reduced as 0.5 equiv of H_2_ are evolved simultaneously (Scheme S3).[^14^, ^27^] Subsequently, **B** is formed (red trace). **B** exhibits a characteristic CO vibration at a frequency of 1982 cm^−1^. The shift of the *A*’ CO vibrational mode of **B** with regard to the one of **1** is characteristic for a 1 ē reduction at each α‐diimine unit and a five‐coordinated metal centre.[^14^, ^28^] Thus, **B** has been assigned previously to [Mn_2_(H_−1_L)(CO)_6_]^−^.^[14]^ We tentatively assume that the ligand is still deprotonated in **B** as this species is formed in the absence of phenol. Upon further reduction a further species **C** appears with CO stretching frequencies at 1902 and 1865 cm^−1^ (Figure S46, green trace). **C** has been observed previously and assigned to [Mn(H_−1_L^3^)(CO)_3_]^3−^, that is a symmetric Mn compound in which each Mn entity is reduced twice.[^14^, ^28^]

The same experiment in the presence of phenol and acetone revealed slight differences in the species formation (Figure 3). Upon reduction of **1** (black trace), **A** is formed (blue trace), and subsequently **B** as major species (red trace, *ν*_CO_=1982, 1902, and 1890 cm^−1^). The characteristic C=O vibration of acetone at a frequency of 1715 cm^−1^ is present at the beginning of the experiment and decreases after formation of **B** under applied potential. Thus, **B** represents the steady state species under catalytic conditions. Towards the end of the experiment, after the C=O band of acetone is vanished, CO stretching vibrations characteristic for **C** appear (Figure S47, green trace).

**Scheme 1:**
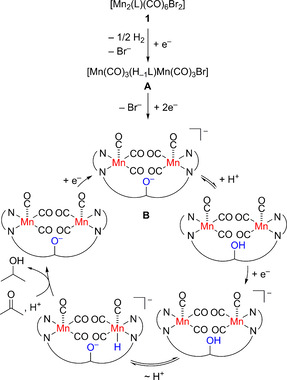
Proposed mechanism for the hydrogenation of acetone using **1**. H_−1_L denotes the ligand deprotonated at the phenol unit.

Since **1** and **A** appear as intermediates during catalysis according to IR‐SEC studies, they were reinvestigated by CV measurements in THF. Scan rate dependent CV data of **1** showed that the peak potentials shift with increasing scan rates indicating coupled chemical reactions after reductions such as OH‐bond breaking and bromide loss as previously observed in DMF (Figure S40).^[14]^ **A** exhibits four reduction processes in THF (*E*_p,c_ of −2.07 V, −2.21 V, −2.66 −2.93 V *ν*=0.1 Vs^−1^, Figure S42). The first reduction process of **1** is not present in **A** supporting the hypothesis that initial reduction of **1** leads indeed to O−H bond breaking and formation of the phenolate complex (Scheme 1, Scheme S3, Figure S42).^[14]^

**Figure 4:**
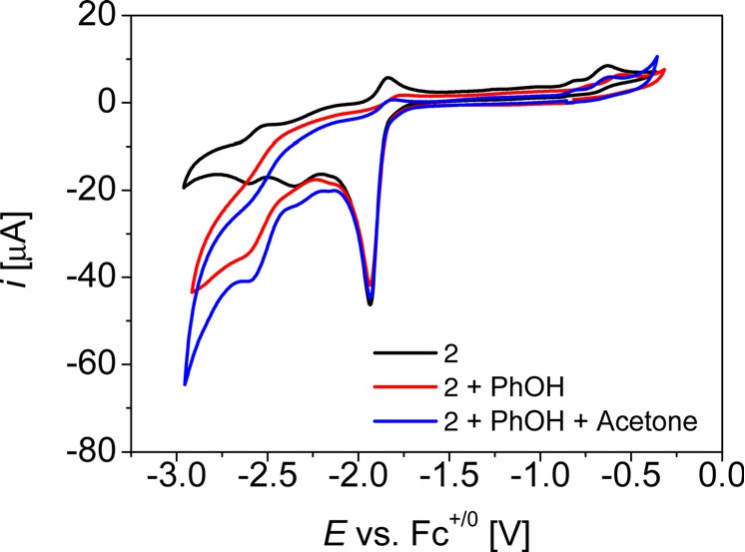
CV data of **2** in THF, *c*_2_=1 mm, *c*_phenol_=10 mm, *c*_acetone_=100 mm, 0.2 m *n*Bu_4_NPF_6_, *ν*=0.1 Vs^−1^.

Subsequently, we investigated the influence of the proton relay on catalysis and utilised **2** as catalyst (Figure S1).^[14]^ Complex **2** has a −OMe function instead of the −OH function. It exhibits an irreversible, multiple electron redox process at −1.93 V in THF (*v*=0.1 Vs^−1^) and further reduction processes at −2.35 V and −2.60 V (Figure 4), similar as previously described in DMF.^[14]^ Upon adding phenol to **2**, a new multiple electron reduction wave appears at −2.60 V, however, it appears at considerably lower potential and less pronounced as in the otherwise same experiment with **1**. Upon adding acetone, the CV trace is very similar to the one without acetone and no new redox processes appear (Figure 4). This indicates that **2** is not an efficient catalyst for hydrogenation of acetone (*ν*=0.1 Vs^−1^). Indeed, when we run a CPE experiment with **2**, acetone, and phenol in THF, considerably less charge was passed than with **1** and considerably smaller amounts of *iso*‐propanol were formed (Figure S5, Figure S43). Isopropanol formation can be rationalised by the large excess of external phenol, which also leads to protonation of the metal centre in an equilibrium reaction, however the external proton source is less efficient and thus, leads to a low reaction rate for catalysis, which highlights the importance of an internal proton relay for catalysis.^[29]^

Taking all measurements together, a preliminary mechanism is proposed (Scheme 1). IR‐SEC studies revealed that the complex keeps the facial tricarbonyl motive during reduction. **B** represents the steady state species under catalysis according to IR‐SEC. In line with this, catalysis appears at the potential of the third reduction of **1**, which is equal to the second reduction of **A**. The reduction of **1** forming **A** and 0.5 equiv of H_2_ requires one electron and further reduction forming **B** requires two electrons. Indeed, electrolysis of **1** at a potential of −2.2 V confirmed that three electrons are consumed upon forming **B** and that H_2_ is evolved simultaneously (Figure S45).[^14^, ^30^] Subsequently, we suggest protonation of **B** in an equilibrium reaction with external phenol. Further reduction leads to the transient formation of a Mn^I^−H entity due to the internal proton shift from the phenol‐OH unit to the metal ion. Based on the calculated p*K*_a_ value of 27 for the similar manganese compound [(L^bpy^)Mn(CO)_3_H] (L^bpy^=2,2’‐bipyridine)^[31]^ and the experimentally determined p*K*_a_ of phenol of 29.14 in MeCN,^[32]^ the proton shift may be best described as an equilibrium reaction between the two species. We assume that such a Mn^I^−H species represents the active species, as such motives were proposed previously in similar thermal protocols with H_2_, though further reduction of the putative MnH species prior transfer cannot be excluded.[^2g^, ^16^, ^33^, ^34^] However, since the coordination sphere of such a species is 6, pre‐coordination of acetone to the Mn−H unit at the same metal centre seems unlikely, and thus we assume H^−^ transfer from the Mn−H unit and of H^+^ from phenol to the ketone.^[34]^ Similar scenario has been suggested recently for the hydrogenation of imines and ketones with manganese(I) complexes and is in line with the observed reaction order of one in phenol and in **1**.[^2g^, ^16^, ^34^] Mezzetti et al. showed in a stochiometric reaction that acetophenone indeed inserts into a Mn−H bond for L^PNP^(CO)_2_Mn−H forming the alcoholate (L^PNP^=bis(2‐(cyclohexyl(methyl)phosphanyl)ethyl)amine).^[34]^

Although, the investigation indicate that catalysis proceeds at only one Mn ion, the reduction of the second Mn(CO)_3_ unit is crucial for catalysis. The corresponding mononuclear complex, **3** (Figure S1), is not active in the electrochemical hydrogenation reaction of ketones. Only a small increase in current was observed in the CV experiment of **3** with phenol at very negative potential, that is, *E*_p_=−2.6 V (*v*=0.1 Vs^−1^) and it does not change, when acetone is added (Figure S44).

## Conclusions

We have developed an electrochemical method for the chemoselective hydrogenation of ketones and aldehydes using a base‐metal catalyst. In a proof of principle study we showed, that the in situ generated hydride species hydrogenates C=O bonds over C=C bonds. The competing hydrogen evolution reaction was supressed successfully as the reactions proceed with moderate to high FE. Detailed mechanistic investigation suggest [Mn_2_(H_−1_L)(CO)_6_]^−14^ as steady state species during catalysis. The internal proton relay represents the key feature to form the putative hydride species as it facilitates proton transfer to the metal centre, which then reacts with the ketone forming the alcohol. **2**, which exhibits an −OMe group instead of −OH is barely active and the same is to say for the mononuclear manganese complex **3** highlighting the importance of the internal proton relay and the two metal centres. The electrochemical protocol provides a safer and green method for the hydrogenation of C=O bonds compared to traditional protocols, as a more redox and energy economic way to the substrate H_2_ is explored and a base metal catalyst was utilised.

## Experimental Section

Complexes **1**, **2** and **3** were synthesised as previously described.^[14]^

### General procedure for the hydrogenation of the ketones and aldehydes

All CPE experiments were carried out inside a glovebox with a closed, divided cell. The counter electrode was separated from the bulk solution by a porous glass frit (P3). Carbon foam was used as working electrode, a Pt spiral as counter electrode, and a Ag/AgNO_3_ electrode as reference electrode. The cathodic compartment was filled with a solution of **1** (1 mm), substrate (20 mm), phenol (150 mm), and benzene (8.2 μL) as internal standard in [D_8_]THF (3 mL). The anodic compartment was filled with a solution of ferrocene (50 mg) in [D_8_]THF (3 mL) as sacrificial reagent. After electrolysis the liquid phase of both compartments of the cell was transferred into a Schlenk flask, which was frozen in liquid nitrogen. The Schlenk flask was connected via a glassbridge to a second flask. A static, high vacuum was applied on the whole system and the frozen electrolysis solution was slowly warmed up, whereas the second flask was cooled with liquid nitrogen in order to condense all volatile compounds. The mixture was analysed by ^1^H NMR spectroscopy afterwards. All alcohols are literature known and thus, the products were identified by comparison with the original data.

## Conflict of interest

The authors declare no conflict of interest.

## Supporting information

As a service to our authors and readers, this journal provides supporting information supplied by the authors. Such materials are peer reviewed and may be re‐organized for online delivery, but are not copy‐edited or typeset. Technical support issues arising from supporting information (other than missing files) should be addressed to the authors.

Supplementary
