# Supplementary material for: Chemoselective Electrochemical Hydrogenation of Ketones and Aldehydes with a Well‐Defined Base‐Metal Catalyst
Source: Chemistry. 2020 Oct 4;26(62):14137–43. doi: 10.1002/chem.202002075 (PMC7702145; doi:10.1002/chem.202002075)
Supplement: Supplementary file 1 — Supplementary [file CHEM-26-14137-s001.pdf]

Chemistry–A European Journal

Supporting Information

Chemoselective Electrochemical Hydrogenation of Ketones and Aldehydes with a Well-Defined Base-Metal Catalyst

Igor Fokin and Inke Siewert*[a]

Supporting Information

Table of Content

Thermodynamic Calculations 2

Hydrogenation of Acetone and Isobutene 2

Solution Free Energies for the Hydride Transfer 2

Thermodynamic Potential of the Proton Reduction in thf 3

Experimental Section 3

General 3

Synthesis 3

Determination of the Faraday Efficiencies for the Reactions 3

NMR data 4

Electrochemical Measurements 11

Controlled Potential Electrolysis Experiments and CV Measurements 12

IR-SEC Experiments 23

References 24

Thermodynamic Calculations

Hydrogenation of Acetone and Isobutene

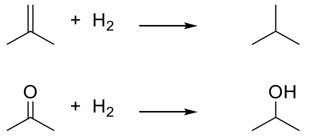

Scheme S 1. Hydrogenation reaction of acetone and isobutene.

Acetone (liquid): $\Delta_f H^0 = -249 \frac{\text{kJ}}{\text{mol}}$,[1] and $S^0 = 200 \frac{\text{J}}{\text{mol}\cdot\text{K}}$[2]

Isopropanol (liquid): $\Delta_f H^0 = -317 \frac{\text{kJ}}{\text{mol}}$,[3] and $S^0 = 180 \frac{\text{J}}{\text{mol}\cdot\text{K}}$[2]

Hydrogen (gas, 1 bar): $S^0 = 131 \frac{\text{J}}{\text{mol}\cdot\text{K}}$[4]

Hydrogenation of acetone: $\Delta_R H^0 = -68 \frac{\text{kJ}}{\text{mol}}$ and $\Delta_R G^0 = -23 \frac{\text{kJ}}{\text{mol}}$

Isobutene (gas, 1 bar): $\Delta_f H^0 = -17.9 \frac{\text{kJ}}{\text{mol}}$, and $S^0 = 294 \frac{\text{J}}{\text{mol}\cdot\text{K}}$[5]

Isobutane (gas, 1 bar): $\Delta_f H^0 = -135.6 \frac{\text{kJ}}{\text{mol}}$[5]

Hydrogenation of isobutene: $\Delta_R H^0 = -118 \frac{\text{kJ}}{\text{mol}}$

Solution Free Energies for the Hydride Transfer

The hydrogenation of acetone by Mn−H is thermodynamically favoured over proton reduction, which is evident from the solution hydricity of H_2 and isopropanol in MeCN (Scheme S 2).

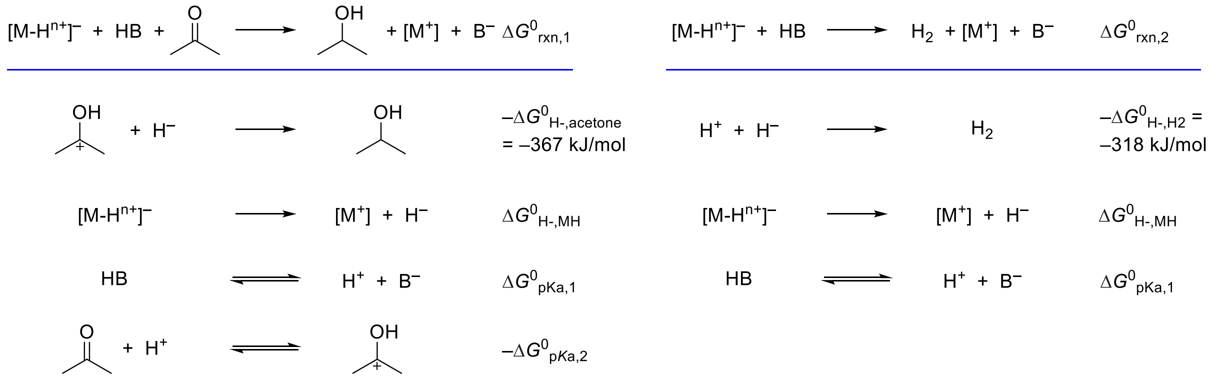

Scheme S 2. Thermodynamic cycle for the formation of isopropanol (left) and H_2 (right) via hydride transfer.

The hydricity of isopropanol in MeCN was calculated previously, the one of H_2 was determined experimentally.[6] The equilibrium of phenol $\Delta_R G^0_{\text{p}K\text{a},1}$ has to be considered in both reactions and the same is to say for the hydricity $\Delta_R G^0_{\text{H}-,\text{MH}}$ of the putative M–H species. The difference in the hydricity of isopropanol and H_2 is about −49 kJ/mol. However, protonation of acetone has to be considered in the latter case, $\Delta_R G^0_{\text{p}K\text{a},2}$, which subtracts about 3 kJ/mol, as the pK_a of protonated acetone in water is −7.2,[7]

which was converted to a pK_a of about 0.6 in acetonitrile by the empirical formula from Ref 8, second group. Thus, the driving force for isopropanol formation is larger than for H_2 formation.

Isopropanol formation: $\Delta_R G^0_{rxn,1} = \Delta_R G^0_{H-,MH} + \Delta_R G^0_{pKa,1} - \Delta_R G^0_{pKa,2} - \Delta_R G^0_{H-,acetone}$

H_2 formation: $\Delta_R G^0_{rxn,2} = \Delta_R G^0_{H-,MH} + \Delta_R G^0_{pKa,1} - \Delta_R G^0_{H-,H2}$

Difference in the driving forces: $\Delta\Delta_R G^0_{rxn} = -\Delta_R G^0_{H-,acetone} - \Delta_R G^0_{pKa,2} + \Delta_R G^0_{H-,H2} \approx -46 \frac{kJ}{mol}$

Thermodynamic Potential of the Proton Reduction in thf

The pK_a of phenol was determined to be 29[9] in thf and the standard potential of the $H^+|H_2$ redox couple was determined to be −0.44 V *vs*. $Fc^{+|0}$ in thf[10].

$$E^\circ_{HA/H_2} = E^\circ_{H^+/H_2} + \frac{RT}{nF}\ln\frac{[Ox]}{[Red]} = E^\circ_{H^+/H_2} - \left(2.303\frac{RT}{nF}\right)pK_a = -2.16\ V$$

Experimental Section

General

Manipulations of air-sensitive reagents were carried out by means of common Schlenk-type techniques involving the use of a dry argon or nitrogen atmosphere or performed in an *MBraun* glovebox. The complexes are light sensitive and thus, are prepared, handled and stored in the dark. All reagents were purchased in chemical grades of 99% or higher and used without further purification.

Synthesis

1, **2** and **3** were synthesised as previously described.[11]

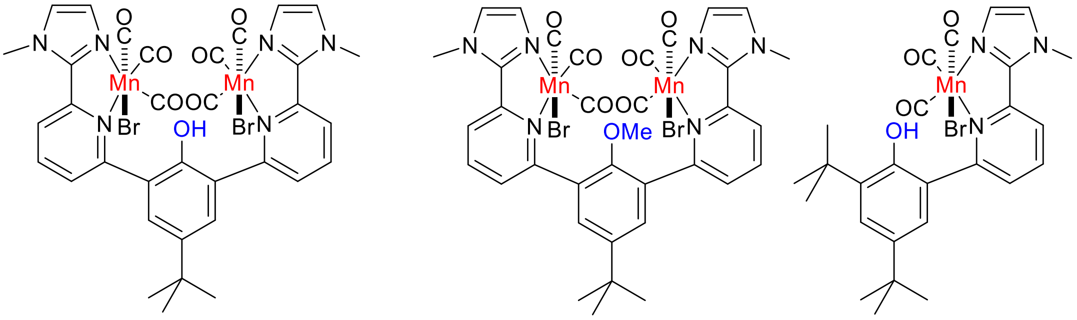

Figure S 1. Structures of the complexes.

Determination of the Faraday Efficiencies for the Reactions

Faraday efficiencies were calculated according to the following equation:

$$FE(\%) = \frac{n_{NMR}}{Q/2F}$$

$n_{nmr} = n_{alcohol}$ as determined by ^{1}H-NMR spectroscopy; Q = injected charge in the experiment – blank charge; F = Faraday constant.

The blank charge counts for the initial reduction step of **1** forming the active species **B**. See main text for detailed discussion of this. The blank charge was determined by applying a potential of −2.2 V to a

solution of **1** in thf (Figure S 2). We subtracted the charge passed at the time at which the individual experiment under catalytic conditions was interrupted.

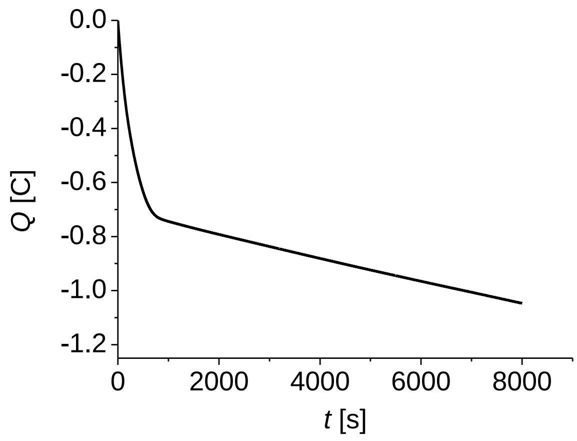

Figure S 2. Charge that was passed for the initial reduction of ***1*** *forming the active species* ***B****. Conditions: thf,* c_1 *= 1 mM, 0.2 M* $^nBu_4NPF_6$*.*

NMR data

General information on the NMR spectroscopy: The ^1H and $^{13}C\{^1H\}$ NMR spectra were recorded with a *Bruker Avance* 300 NMR spectrometer (^1H 300 MHz, ^{13}C 75.4 MHz) with [D$_8$]-thf as the solvent at 25 °C. The ^1H and $^{13}C\{^1H\}$ NMR spectra were calibrated against the residual protons and natural-abundance ^{13}C resonances of the deuterated solvents ([D$_8$]-thf δ_H = 3.58 ppm, δ_C = 67.57 ppm).

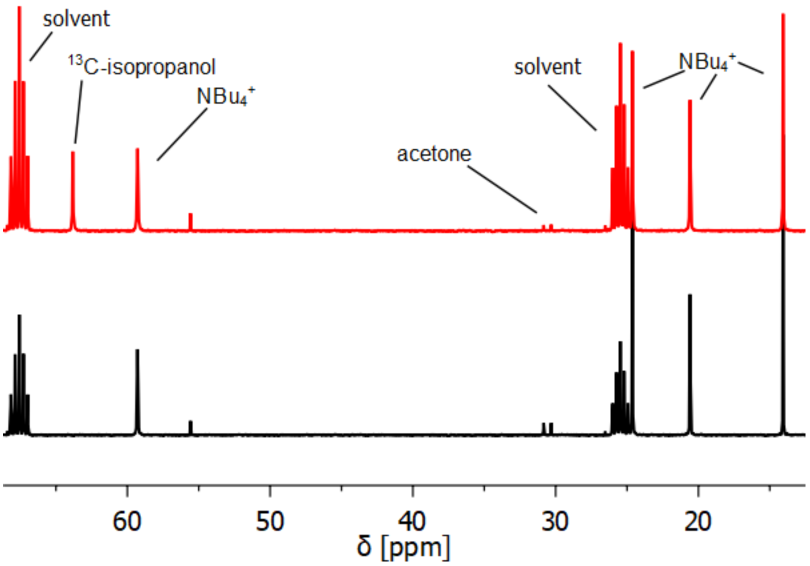

Figure S 3. ^{13}*C-NMR of the reaction mixture after CPE experiments of* $CH_3{-}^{13}CO{-}CH_3$ *and phenol with* ***1*** *(red) and in absence of* ***1*** *(black).*

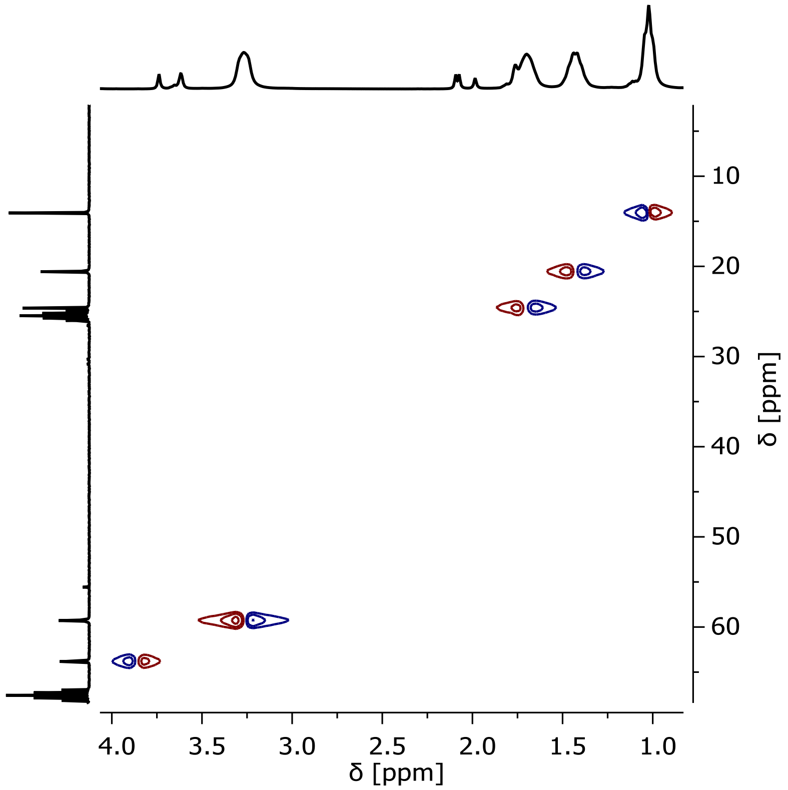

Figure S 4. HSQC-NMR of the reaction mixture after CPE experiment of $CH_3{-}^{13}CO{-}CH_3$ and phenol with ***1****.*

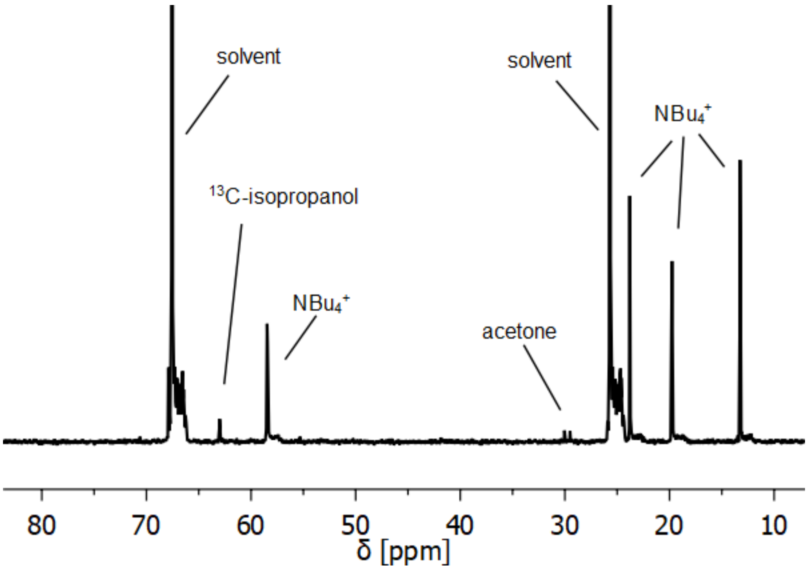

Figure S 5. ^{13}C-NMR of the reaction mixture after CPE experiments of $CH_3{-}^{13}CO{-}CH_3$ and phenol with ***2****, details on the CPE experiment in Figure S 43.*

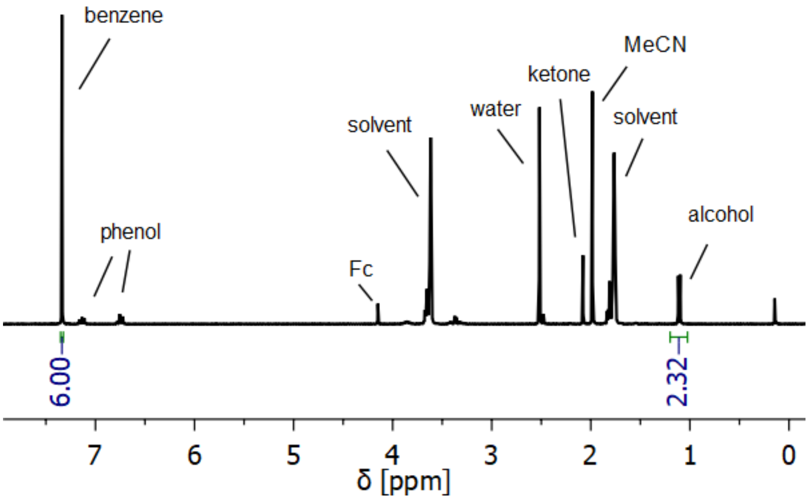

Figure S 6. ^{1}H-NMR of the reaction mixture after CPE experiment and condensation of acetone and phenol with 1 in [D8]-thf. Details on the CPE experiments can be found in Figure 17.

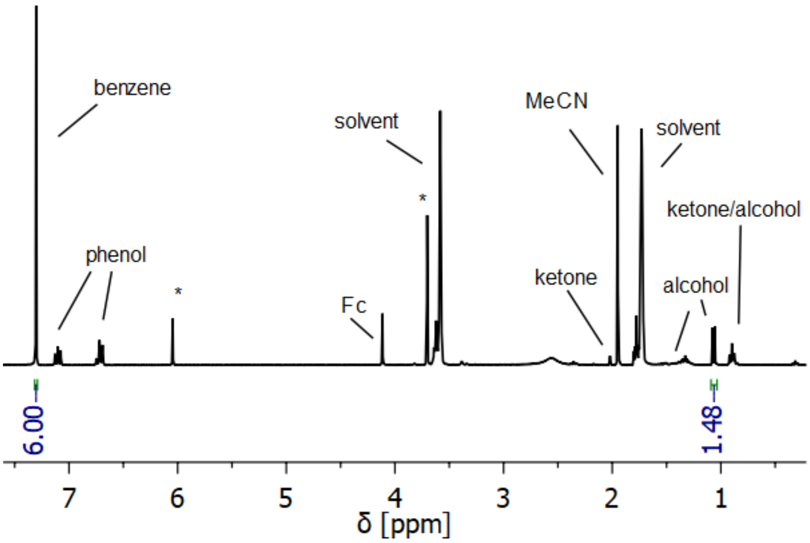

*Figure S 7. ^{1}H-NMR of the reaction mixture after CPE experiment and condensation of 2-pentanone and phenol with **1** in [D$_8$]-thf. Details on the CPE experiments can be found in Figure 21. Peaks marked with asterisk (*) belong to 1,3,5-trimethoxybenzene, which was used as NMR-standard in earlier experiments.*

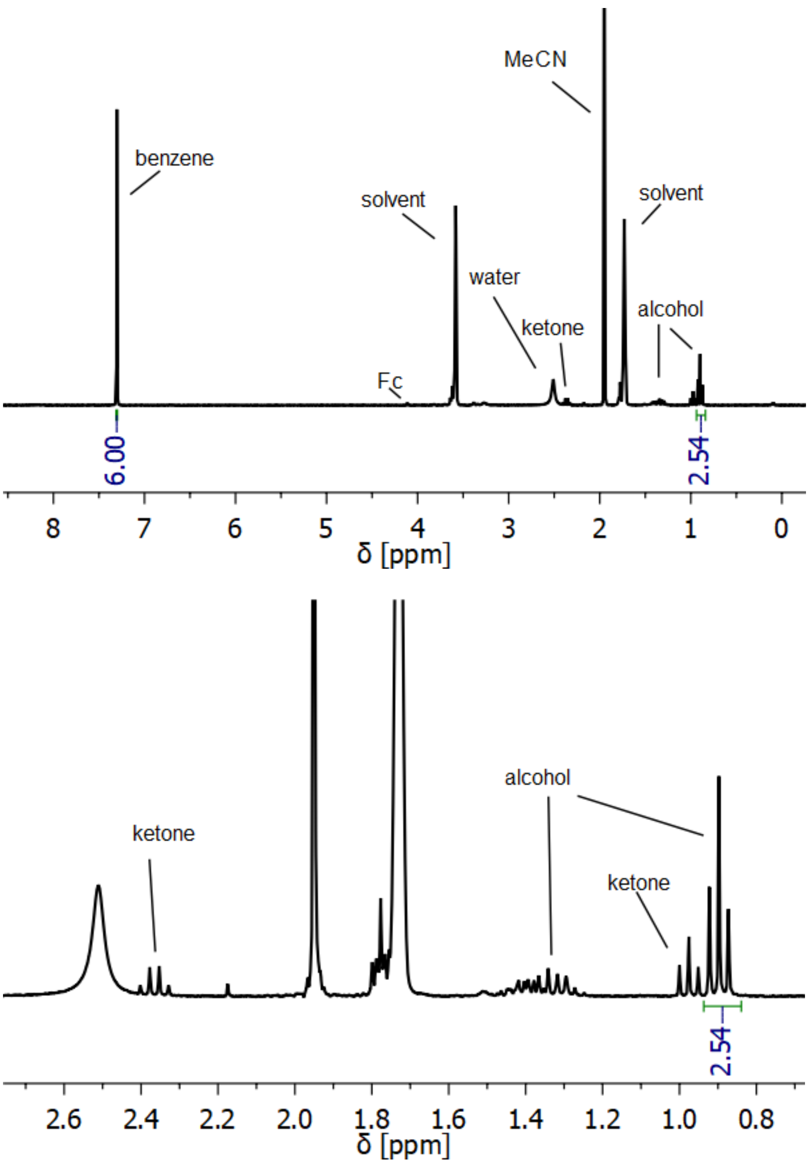

Figure S 8. ^{1}H-NMR spectra of the reaction mixture after CPE experiment and condensation of 3-pentanone and phenol with ***1*** *in $[D_8]$-thf. Details on the CPE experiments can be found in Figure 19.*

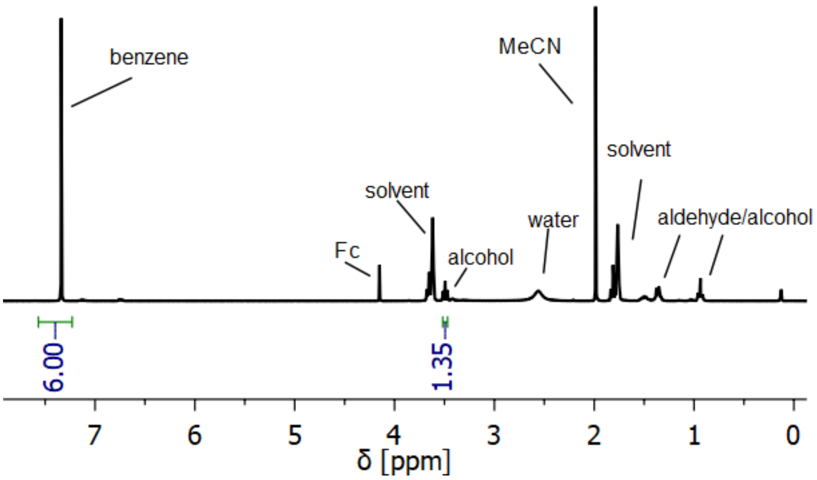

Figure S 9. [1]*H-NMR of the crude mixture after CPE experiment and condensation of pentanal and phenol with* ***1*** *in [D*$_8$*]-thf. Details on the CPE experiments can be found in Figure 23.*

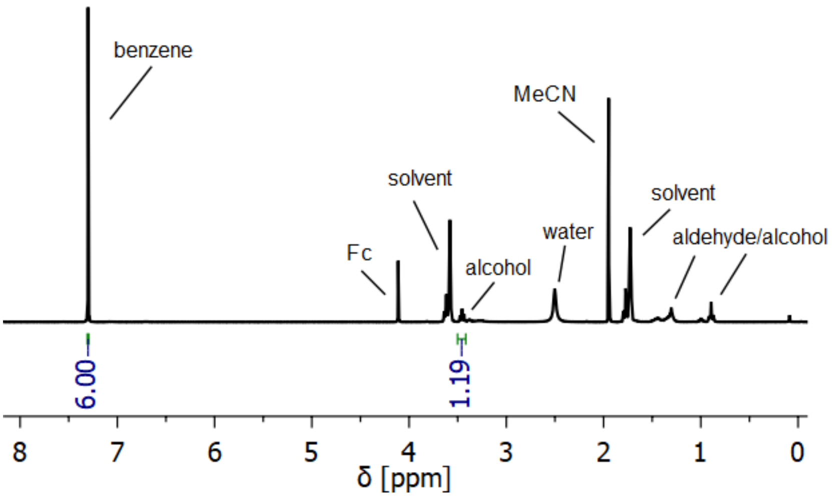

Figure S 10. [1]*H-NMR of the reaction mixture after CPE experiment and condensation of hexanal and phenol with* ***1*** *in [D*$_8$*]-thf. Details on the CPE experiments can be found in Figure 25.*

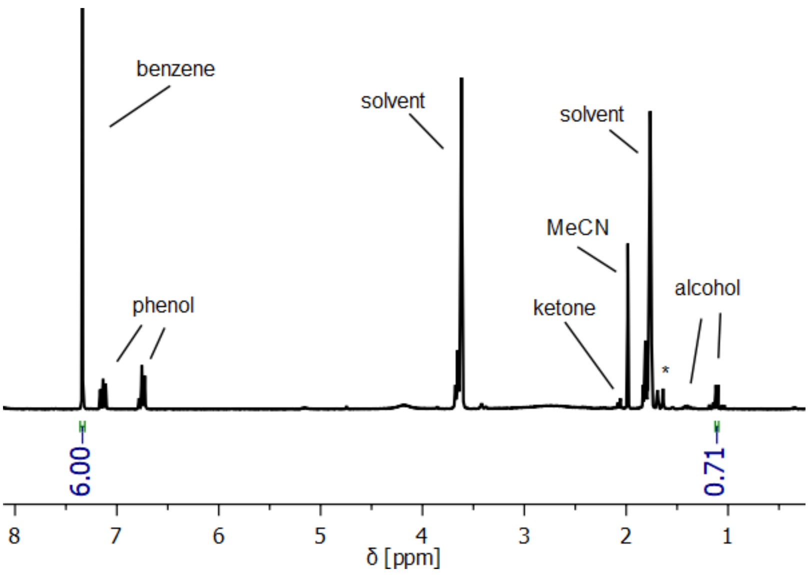

Figure S 11. ^{1}H-NMR of the reaction mixture after CPE experiment and condensation of 6-methyl-5-hept-2-one and phenol with **1** *in [D$_8$]-thf. Details on the CPE experiments can be found in Figure 27. Peaks marked with asterisk (*) belong to ketone/alcohol.*

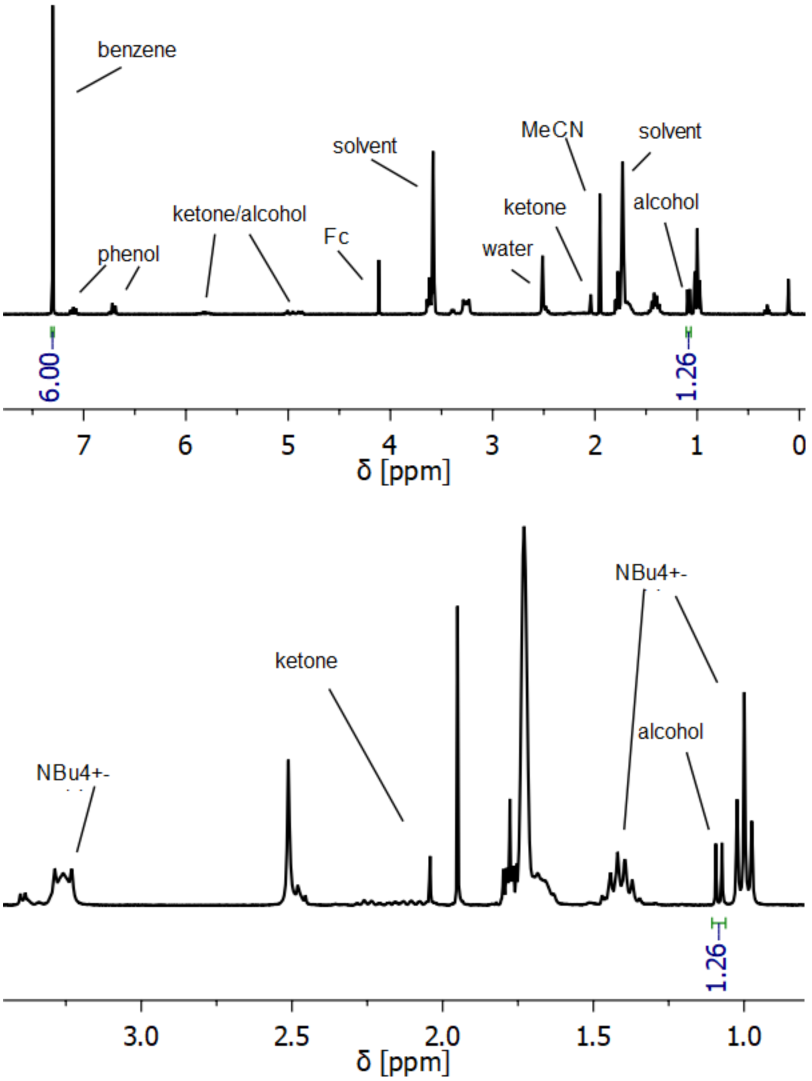

Figure S 12.. ^{1}H-NMR of the reaction mixture after CPE experiment and condensation of 5-hexen-2-one and phenol with ***1*** *in [D$_8$]-thf. Details on the CPE experiments can be found in Figure 29.*

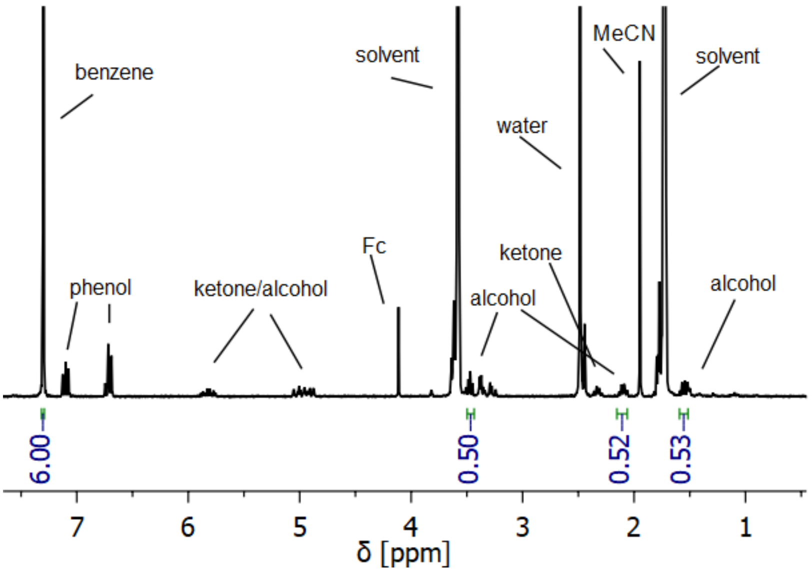

*Figure S 13. ^{1}H-NMR of the reaction mixture after CPE experiment and condensation of 4-pentenal and phenol with **1** in [D$_8$]-thf. Details on the CPE experiments can be found in Figure 31.*

Electrochemical Measurements

General information on the electrochemistry: All CV measurements have been conducted in brown glassware with a glassy carbon (GC) working electrode (diameter 3 mm, *ALS* or *CHI*), a platinum wire counter electrode (*Chempur*), and an Ag/AgNO$_3$ reference electrode. All data were referenced versus the Fc$^{+|0}$ redox couple by adding ferrocene at the end of the measurements. The measurements have been conducted with *Gamry Reference 600*, or *600+* potentiostats. i*R* compensation was performed by the positive feedback method, which is implemented in the *PHE200* software of Gamry.

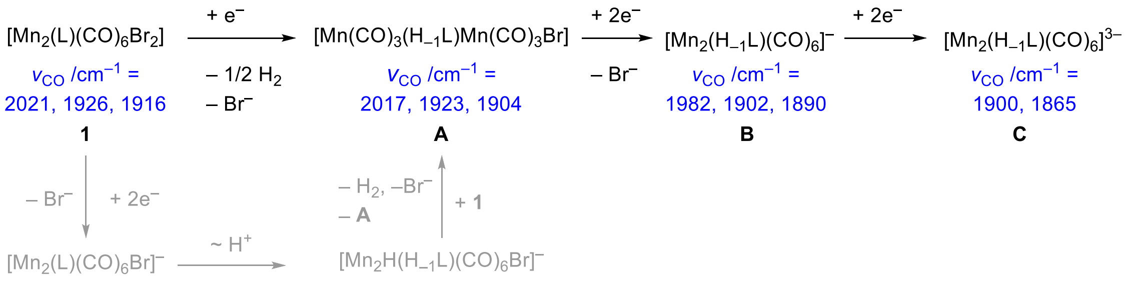

*Scheme S 3. Reduction chemistry of **1** in thf in the absence of substrates.[11] The species depicted in grey have not been observed spectroscopically. The CO stretching frequencies of the transient reduced species are shown in blue.*

Controlled Potential Electrolysis Experiments and CV Measurements

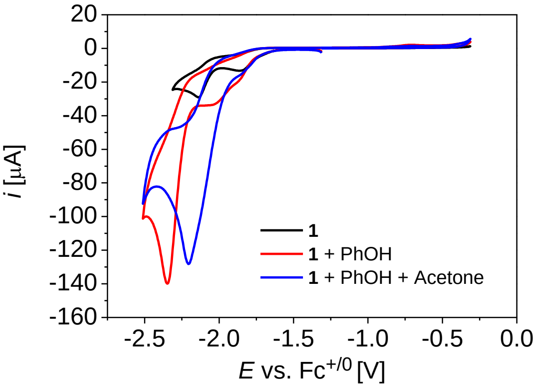

*Figure S 14. CV data of **1** in CH_2Cl_2, c_1 = 1 mM, c_{phenol} = 10 mM, $c_{acetone}$ = 100 mM, 0.1 M $^nBu_4NPF_6$, v = 0.1 Vs^{-1}.*

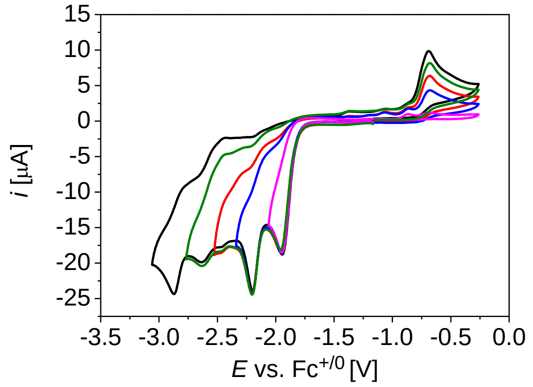

*Figure S 15. CV data of **1** in thf, c_1 ~ 1 mM, 0.2 M $^nBu_4NPF_6$, v = 0.1 Vs^{-1}.*

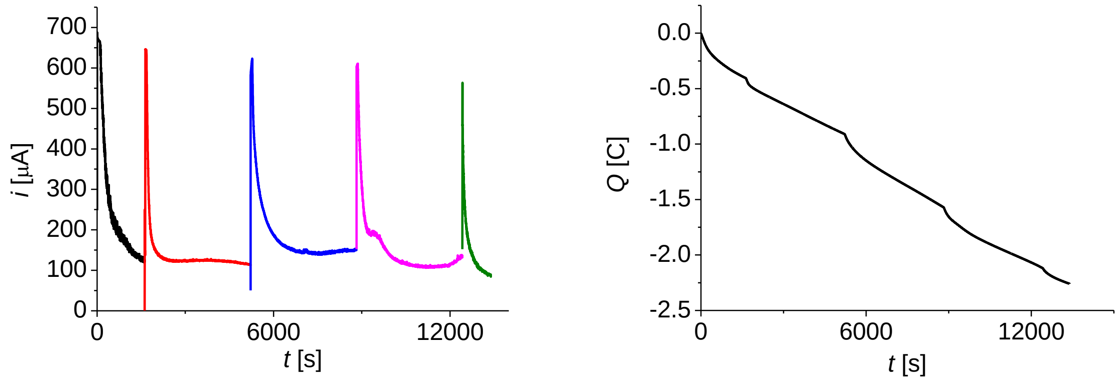

Figure S 16. Current vs. time plot for the controlled electrolysis experiment in CH_2Cl_2. The electrode was polished during the experiment as indicated by the colour change of the trace. Conditions: c_1 = 1 mM, c_{phenol} = 100 mM, $c_{acetone}$ = 100 M, 0.1 M $^nBu_4NPF_6$, E^{appl} = −2.2 V.

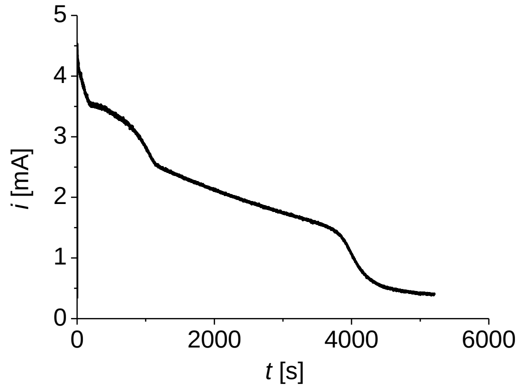

Figure S 17. Current vs. time plot for the CPE experiment of acetone in thf. Conditions: c_1 = 1 mM, c_{phenol} = 150 mM, $c_{acetone}$ = 20 mM, 0.2 M $^{n}Bu_4NPF_6$, E^{appl} = −2.2 V.

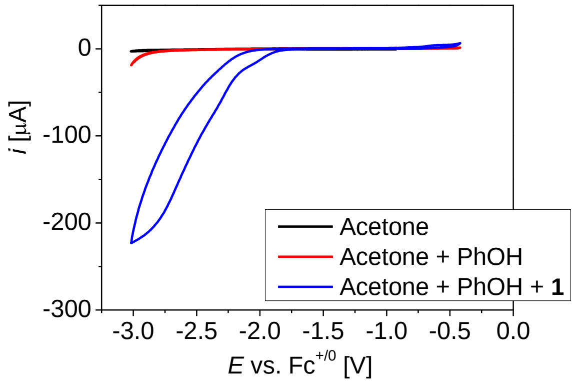

*Figure S 18. CV data of acetone, phenol and **1** in thf under the conditions of the CPE, c_1 = 1 mM, $c_{carbonyl}$ = 20 mM, c_{phenol} = 150 mM, 0.2 M $^{n}Bu_4NPF_6$, v = 0.1 Vs^{-1}.*

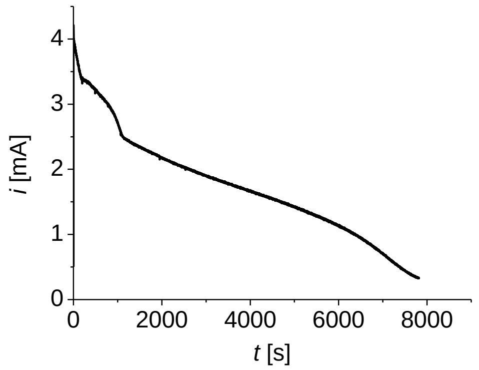

Figure S 19. Current vs. time plot for the CPE experiment of pentan-3-one in thf under the conditions of the CPE experiment, c_1 = 1 mM, c_{phenol} = 150 mM, $c_{pentan\text{-}3\text{-}one}$ = 20 mM, 0.2 M $^{n}Bu_4NPF_6$, E^{appl} = −2.2 V.

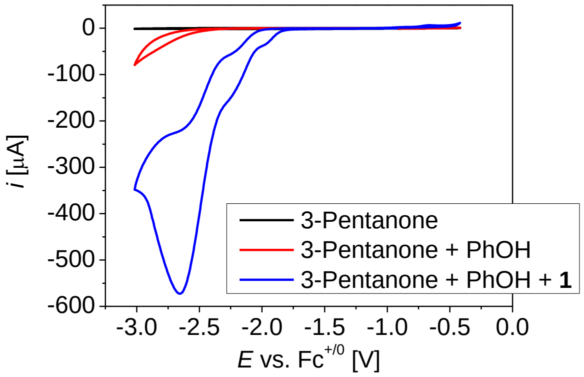

Figure S 20. CV data of pentan-3-one, phenol and ***1*** *in thf under the conditions of the CPE,* c_1 *= 1 mM,* $c_{carbonyl}$ *= 20 mM,* c_{phenol} *= 150 mM, 0.2 M* $^{n}Bu_4NPF_6$*,* v *= 0.1* Vs^{-1}*.*

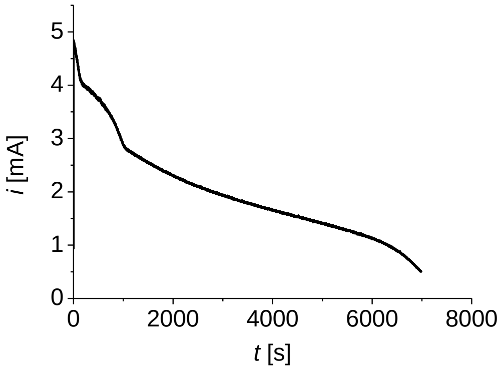

Figure S 21. Current vs. time plot for the CPE experiment of pentan-2-one in thf. Conditions: c_1 *= 1 mM,* c_{phenol} *= 150 mM,* $c_{pentan\text{-}2\text{-}one}$ *= 20 mM, 0.2 M* $^{n}Bu_4NPF_6$*,* E^{appl} *= −2.2 V.*

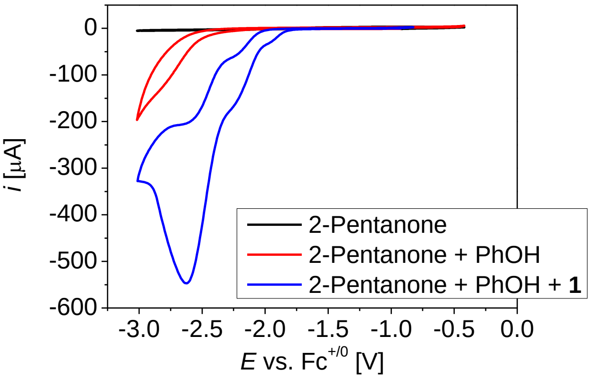

Figure S 22. CV data of 2-pentanone, phenol and ***1*** *in thf under the conditions of the CPE,* c_1 *= 1 mM,* $c_{carbonyl}$ *= 20 mM,* c_{phenol} *= 150 mM, 0.2 M* $^{n}Bu_4NPF_6$*,* v *= 0.1* Vs^{-1}*.*

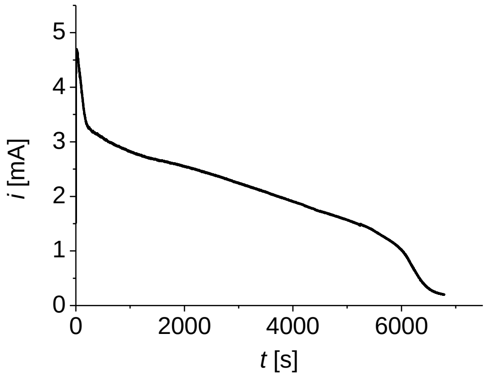

Figure S 23. Current vs. time plot for the CPE experiment of pentanal in thf. Conditions: $c_1 = 1$ *mM,* $c_{phenol} = 150$ *mM,* $c_{pentanal} = 20$ *mM, 0.2 M* $^nBu_4NPF_6$*,* $E^{appl} = -2.1$ *V.*

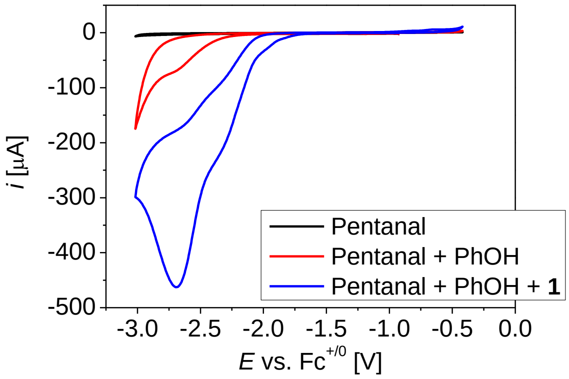

Figure S 24. CV data of pentanal, phenol and ***1*** *in thf under the conditions of the CPE,* $c_1 = 1$ *mM,* $c_{carbonyl} = 20$ *mM,* $c_{phenol} = 150$ *mM, 0.2 M* $^nBu_4NPF_6$*,* $v = 0.1$ Vs^{-1}*.*

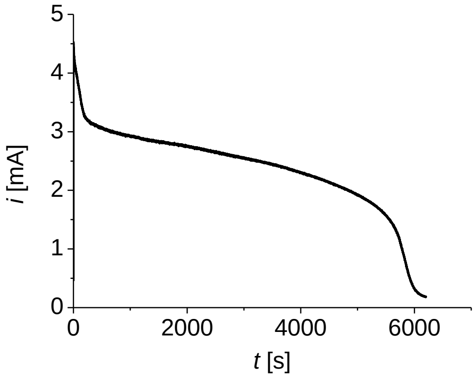

Figure S 25. Current vs. time plot for the CPE experiment of hexanal in thf. Conditions: $c_1 = 1$ *mM,* $c_{phenol} = 150$ *mM,* $c_{hexanal} = 20$ *mM, 0.2 M* $^nBu_4NPF_6$*,* $E^{appl} = -2.2$ *V.*

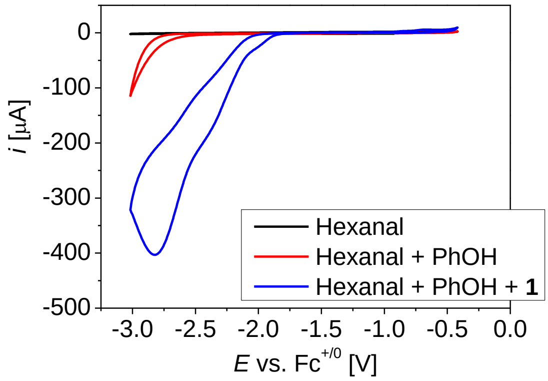

*Figure S 26. CV data of hexanal, phenol and **1** in thf under the conditions of the CPE, c_1 = 1 mM, $c_{carbonyl}$ = 20 mM, c_{phenol} = 150 mM, 0.2 M ^{n}Bu$_4$NPF$_6$, v = 0.1 Vs^{-1}.*

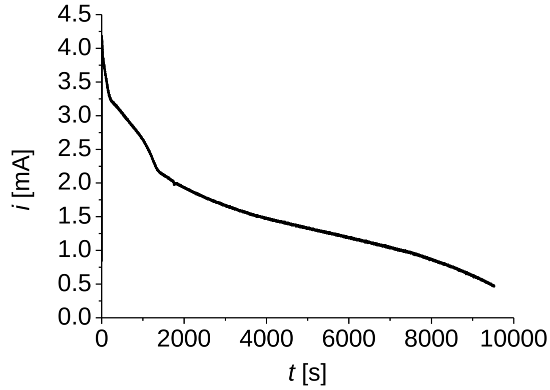

Figure S 27. Current vs. time plot for the CPE experiment of 6-methylhept-5-en-2-one in thf. Conditions: c_1 = 1 mM, c_{phenol} = 150 mM, $c_{6\text{-methylhept-5-en-2-one}}$ = 20 mM, 0.2 M ^{n}Bu$_4$NPF$_6$, E^{appl} = −2.2 V.

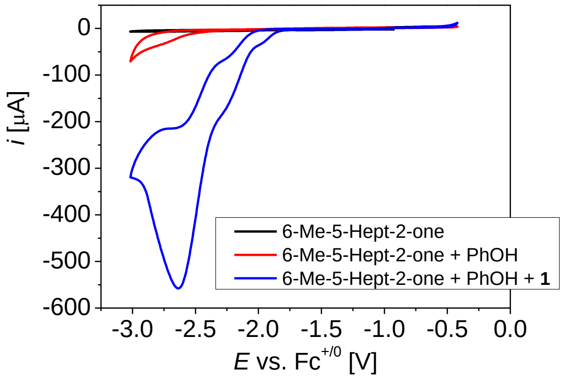

*Figure S 28. CV data of 6-methyl-5-hept-2-one, phenol and **1** in thf under the conditions of the CPE, c_1 = 1 mM, $c_{carbonyl}$ = 20 mM, c_{phenol} = 150 mM, 0.2 M ^{n}Bu$_4$NPF$_6$, v = 0.1 Vs^{-1}.*

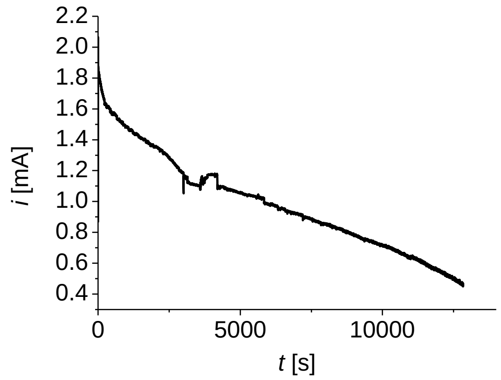

Figure S 29. Current vs. time plot for the CPE experiment of hex-5-en-2-one in thf. Conditions: c_1 *= 1 mM,* c_{phenol} *= 150 mM,* $c_{hex\text{-}5\text{-}en\text{-}2\text{-}one}$ *= 20 mM, 0.2 M* $^{n}Bu_4NPF_6$*,* E^{appl} *= −2.2 V.*

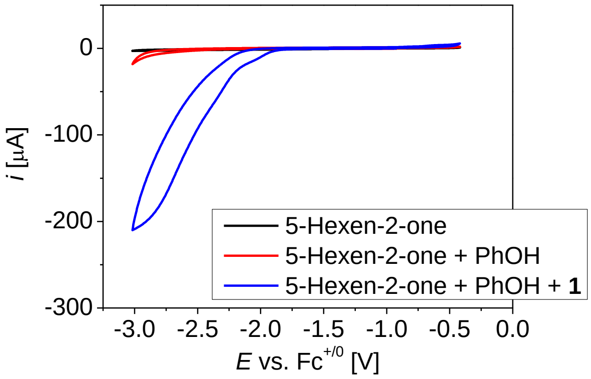

Figure S 30. CV data of hex-5-en-2-one, phenol and ***1*** *in thf under the conditions of the CPE,* c_1 *= 1 mM,* $c_{carbonyl}$ *= 20 mM,* c_{phenol} *= 150 mM, 0.2 M* $^{n}Bu_4NPF_6$*, v = 0.1* Vs^{-1}*.*

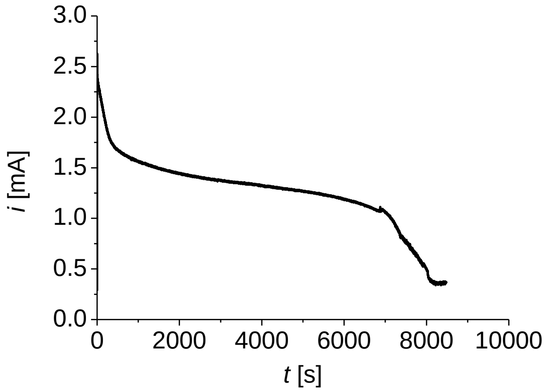

Figure S 31. Current vs. time plot for the CPE experiment of pent-4-enal in thf. Conditions: c_1 *= 1 mM,* c_{phenol} *= 150 mM,* $c_{pent\text{-}4\text{-}enal}$ *= 20 mM, 0.2 M* $^{n}Bu_4NPF_6$*,* E^{appl} *= −2.2 V.*

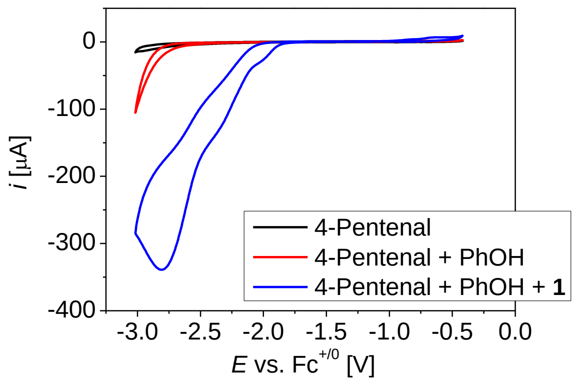

*Figure S 32. CV data of pent-4-enal, phenol and **1** in thf under the conditions of the CPE, c_1 = 1 mM, $c_{carbonyl}$ = 20 mM, c_{phenol} = 150 mM, 0.2 M ^{n}Bu$_4$NPF$_6$, v = 0.1 Vs^{-1}.*

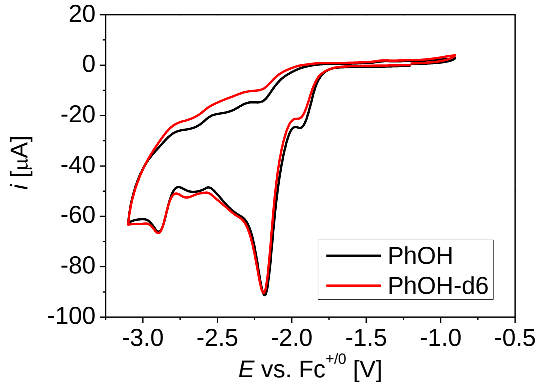

*Figure S 33. CV measurements of acetone, phenol, and **1** in thf, c_1 = 1 mM, c_{phenol} = 10 mM, $c_{acetone}$ = 100 mM, 0.2 M ^{n}Bu$_4$NPF$_6$, v = 0.1 Vs^{-1}.*

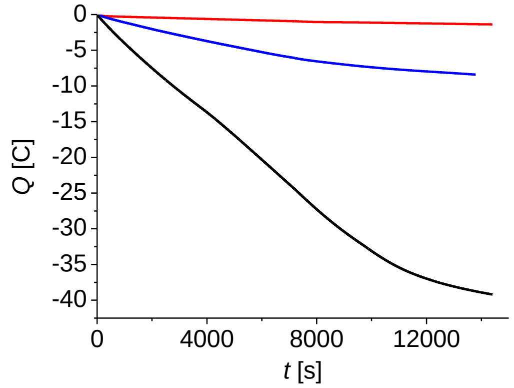

*Figure S 34. Charge vs. time plot for the CPE experiments of **1** in thf using phenol-OH (black) and phenol-OD (blue) compared to an electrolysis experiment in absence of **1** (red). Conditions: c_1 = 1 mM, c_{phenol} = 300 mM, $c_{acetone}$ = 100 mM, 0.2 M ^{n}Bu$_4$NPF$_6$.*

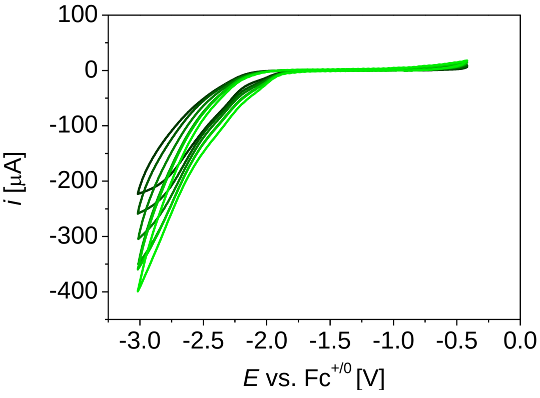

Figure S 35. Scan rate dependent CV data of ***1,*** *phenol and acetone in thf, c = 1 mM,* $c_{acetone}$ *= 20 mM,* c_{phenol} *= 150 mM, 0.2 M* $^{n}Bu_4NPF_6$*,* v *= 0.1, 0.2, 0.4, 0.8, 1, 2* Vs^{-1}*.*

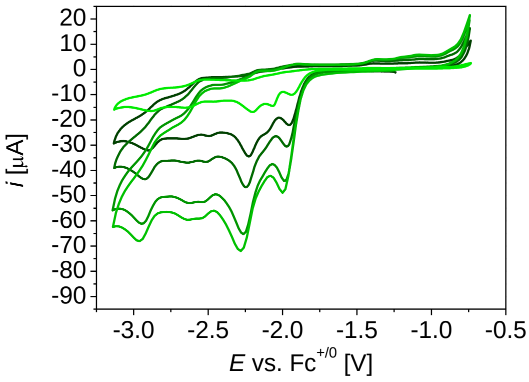

Figure S 36. Scan rate dependent CV data of ***1*** *and acetone in thf,* c_1 *= 1 mM,* $c_{acetone}$ *= 10 mM, 0.2 M* $^{n}Bu_4NPF_6$*,* v *= 0.1, 0.2, 0.4, 0.8, 1* Vs^{-1}*.*

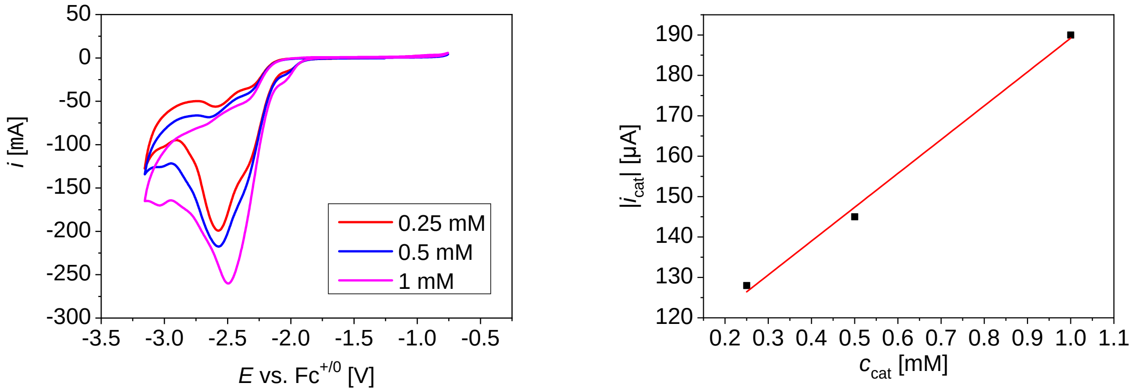

Figure S 37. Left: CV measurements of acetone, phenol, and varying concentrations of catalyst in thf, c_{phenol} *= 50 mM,* $c_{acetone}$ *= 50 mM, 0.2 M* $^{n}Bu_4NPF_6$*,* v *= 0.1* Vs^{-1}*. Right: Plot of the maximum catalytic current for the hydrogenation reaction vs. catalyst concentration, the red line represents the fit.*

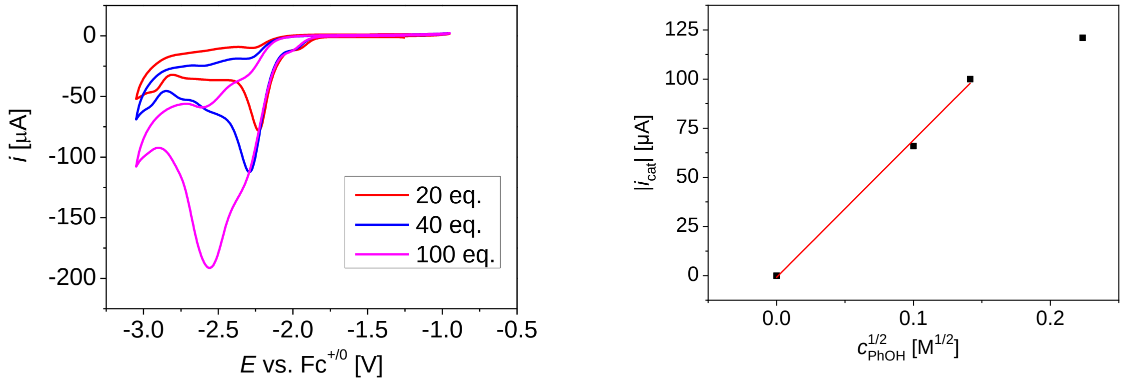

*Figure S 38. Left: CV measurements of **1**, acetone, and varying concentrations of phenol in thf, c_1 = 0.5 mM, $c_{acetone}$ = 50 mM, 0.2 M ^{n}Bu$_4$NPF$_6$, v = 0.1 Vs^{-1}. Right: Plot of the maximum catalytic current for the hydrogenation reaction vs. the square root of the phenol concentration, the red line represents the fit.*

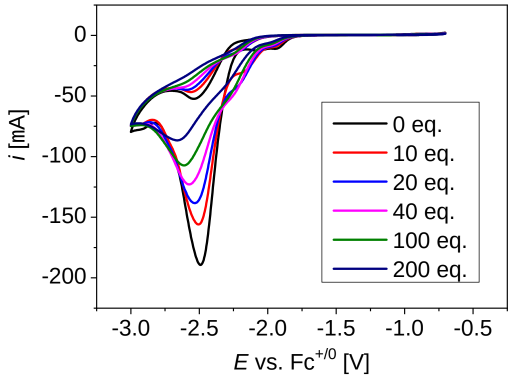

*Figure S 39. CV measurements of **1**, phenol, and varying concentrations of acetone in thf, c_1 = 0.5 mM, c_{phenol} = 50 mM, 0.2 M ^{n}Bu$_4$NPF$_6$, v = 0.05 Vs^{-1}.*

In the presence of large excess of phenol, a prominent catalytic wave appears at around −2.4 V, which belongs to the hydrogen evolution reaction. The wave decreases with increasing amounts of acetone indicating that the reaction is successfully supressed. However, since the wave belonging to the hydrogenation reaction of acetone and the one belonging to the hydrogen evolution reaction overlap, the current cannot be reliable determined under pseudo first order conditions for acetone, i.e. in the presence of large excess of phenol.

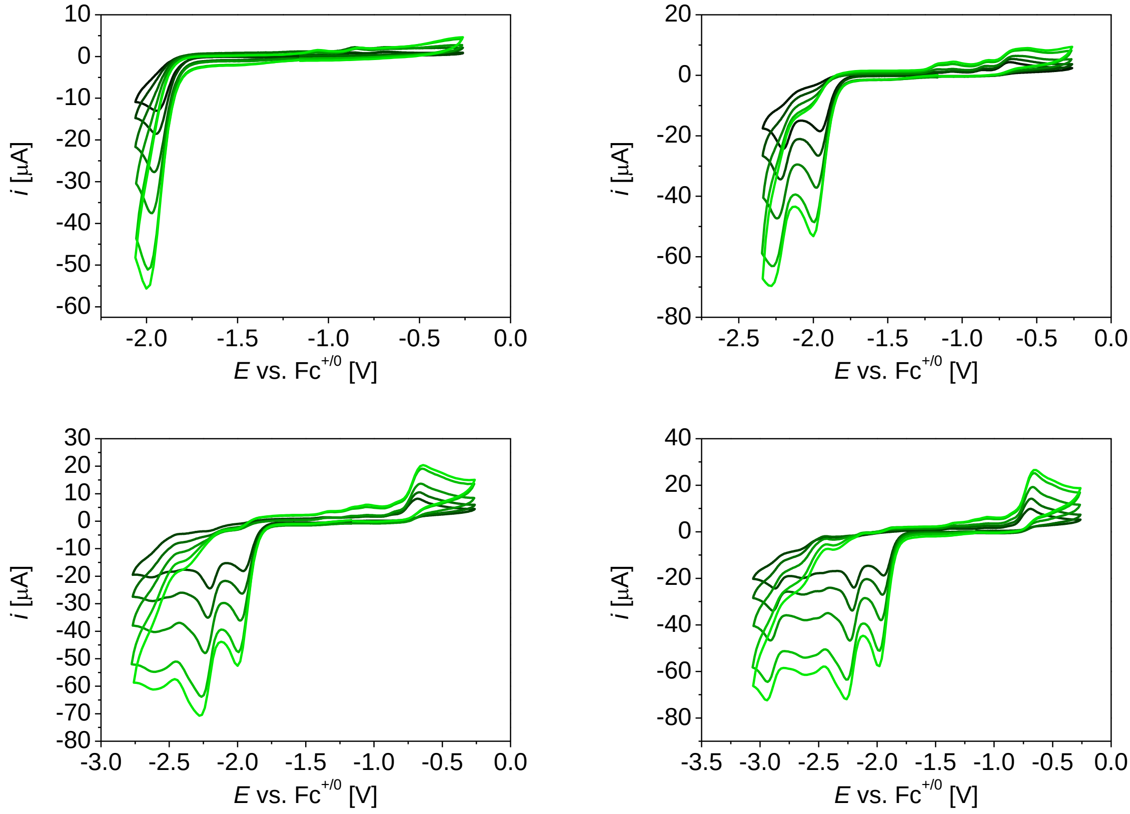

*Figure S 40. Scan rate dependent CV data of **1** in thf with different return potentials, c_1 = 1 mM, 0.2 M ^{n}Bu$_4$NPF$_6$, v = 0.1, 0.2, 0.4, 0.8, 1 Vs^{-1}.*

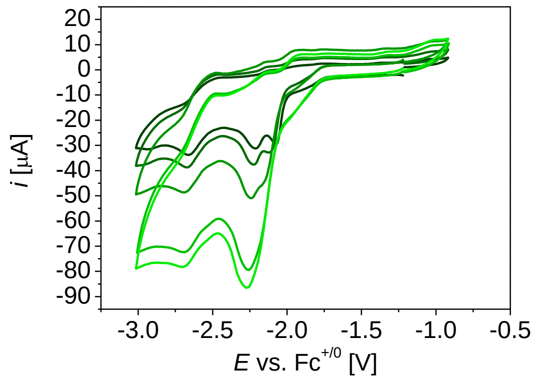

*Figure S 41. CV data of **1** + 1 equiv KO^{t}Bu (= **A**) in thf, c_1 = 1 mM, 0.2 M ^{n}Bu$_4$NPF$_6$, v = 0.1, 0.2, 0.4, 0.8, 1 Vs^{-1}.*

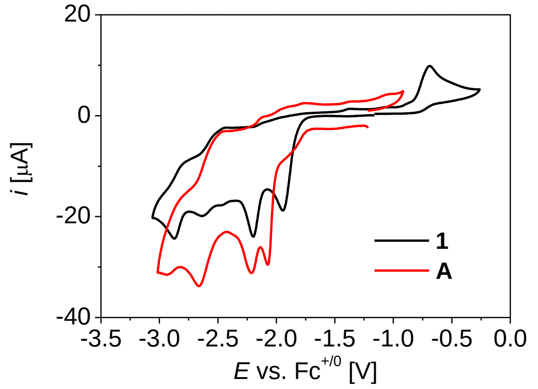

Figure S 42. Overlay of the CV data of ***1*** *and* ***1*** *+ 1 equiv KO^{t}Bu (=* ***A****) in thf, c = 1 mM, 0.2 M ^{n}Bu$_4$NPF$_6$, v = 0.1 Vs^{-1}.*

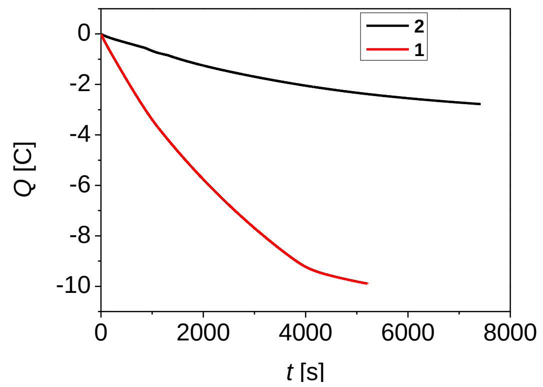

Figure S 43. Charge vs. time plot for the CPE experiment of ***1*** *and* ***2*** *with phenol and acetone in thf. Conditions: c_{cat} = 1 mM, c_{phenol} = 150 mM, $c_{acetone}$ = 20 mM, 0.2 M ^{n}Bu$_4$NPF$_6$, E^{appl} = –2.2 V.*

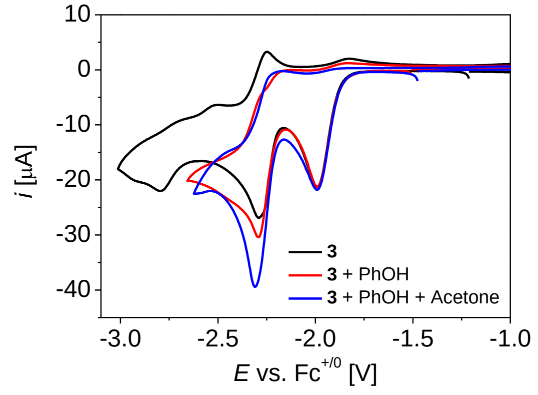

Figure S 44. CV data of ***3*** *in thf, c_3 = 1 mM, c_{phenol} = 10 mM, $c_{acetone}$ = 100 mM, 0.2 M ^{n}Bu$_4$NPF$_6$, v = 0.1 Vs^{-1}.*

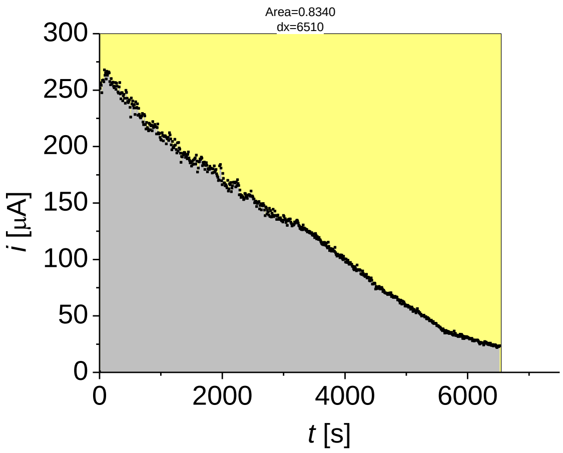

*Figure S 45. Current vs. time plot for the CPE experiment of **1** in thf at –2.2 V, c_1 = 1 mM, 0.2 $^{n}Bu_4NPF_6$.*

The experiment was stopped, when the current dropped down to about 10 % of the initial current. The charge passed counts for 2.7 electrons.

IR-SEC Experiments

IR-SEC experiments were conducted in an OTTLE cell.[12] The cell is equipped with a platinum mesh working electrode in the optical path, a pseudo-Ag-reference, and a platinum counter electrode. The IR spectra were recorded with a *Bruker Invenio-R* spectrometer equipped with a MCT-Detector (15.500 - 350 cm^{-1}). The scan rate for the linear sweep voltammogram was 0.0025 Vs^{-1} and IR-spectra were recorded every twelve seconds, that is an IR-spectrum was recorded every 30 mV.

We investigated **1** by IR-SEC in the absence of acetone and phenol in thf. The species distribution was were very similar to the species distribution previously observed in dmf (Scheme S 3).[11] For a detailed discussion and assignment of the individual species we refer to this paper.

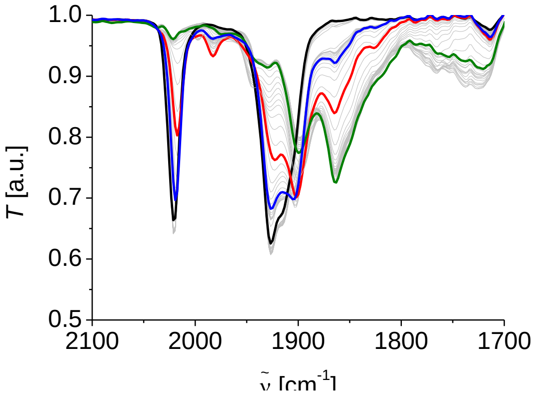

*Figure S 46. Linear sweep IR-SEC of **1** in thf, I = 0.2 M $^{n}Bu_4NPF_6$. The black spectrum denotes the start spectrum, the blue one after initial reduction and the green one the final spectrum.*

We conducted an additional IR-SEC experiment of **1**, phenol and acetone and applied the potential for a longer time. Towards the end of the experiment, after the C=O band of acetone is vanished, CO stretching vibrations characteristic for **C** appear as in the absence of the substrate (Figure S 47, green trace).

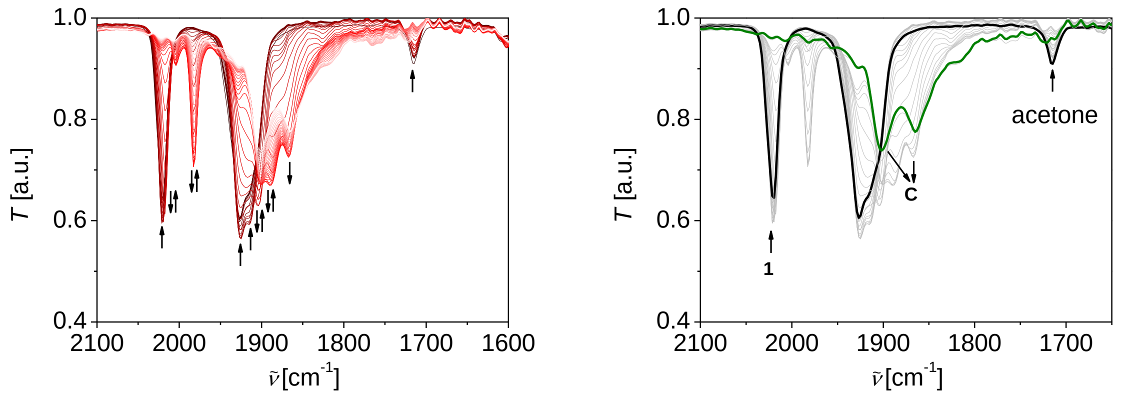

*Figure S 47. Two different presentations of the linear sweep IR-SEC experiment of **1** in thf in the presence of acetone and [D_6]-phenol, I = 0.2 M $^nBu_4NPF_6$. Left: The start spectrum is depicted in dark red and the spectra are coloured lighter with increasing time. Right: black represents the start spectrum and the green spectrum represents the final species after all acetone is consumed and the potential was set to more negative potentials than the catalytic wave.*

References

[1] K. B. Wiberg, L. S. Crocker, K. M. Morgan, *J. Am. Chem. Soc.* **1991**, *113*, 3447-3450.
[2] G. S. Parks, K. K. Kelley, H. M. Huffman, *J. Am. Chem. Soc.* **1929**, *51*, 1969-1973.
[3] J. Chao, F. D. Rossini, *J. Chem. & Eng. Data* **1965**, *10*, 374-379.
[4] Chase, M.W., Jr., *NIST-JANAF Themochemical Tables, 4th Edition, J. Phys. Chem. Ref. Data, Monograph 9*, **1998**, p. 1310.
[5] P. Linstrom, *NIST Chemistry WebBook, NIST Standard Reference Database 69*, 1997, https://webbook.nist.gov, retrieved 02.03.2020.
[6] J. T. Muckerman, P. Achord, C. Creutz, D. E. Polyansky, E. Fujita, *Proc. Natl. Acad. Sci. USA* **2012**, *109*, 15657-15662.
[7] H. J. Campbell, J. T. Edward, *Can. J. Chem.* **1960**, *38*, 2109-2116.
[8] E. Rossini, A. D. Bochevarov, E. W. Knapp, *ACS Omega* **2018**, *3*, 1653-1662.
[9] D. Barrón, J. Barbosa, *Anal. Chim. Acta* **2000**, *403*, 339-347.
[10] S. Daniele, P. Ugo, G.-A. Mazzocchin, G. Bontempelli, *Anal. Chim. Acta* **1985**, *173*, 141-148.
[11] I. Fokin, A. Denisiuk, C. Würtele, I. Siewert, *Inorg. Chem.* **2019**, *58*, 10444-10453.
[12] M. Krejcik, M. Danek, F. Hartl, *J. Electroanal. Chem.* **1991**, *317*, 179-187.
